# The effect of eye health system strengthening on the productivity of services: a before-and-after evaluation conducted in seven counties in Kenya

**DOI:** 10.3389/frhs.2026.1901542

**Published:** 2026-07-14

**Authors:** Joyce Koech, Jefitha Karimurio, Mary B. Adam, Babar Qureshi, Susann Fritzsche, Faith Kasoly

**Affiliations:** 1 Institute of Healthcare Management, Strathmore Business School, Nairobi, Kenya; 2 Inclusive Health Initiative, Christian Blind Mission, Nairobi, Kenya; 3 Department of Ophthalmology, University of Nairobi, Nairobi, Kenya; 4 Paediatrics and Community Health Unit, Kijabe Hospital, Kiambu, Kenya; 5 Inclusive Health Initiative, Christian Blind Mission, Cambridge, United Kingdom; 6 Inclusive Health Initiative, Christian Blind Mission, Bensheim, Germany

**Keywords:** eye health, health service, health system strengthening (HSS), health workforce, intervention, ophthalmic equipment

## Abstract

**Introduction:**

Health system strengthening is essential for achieving universal eye health coverage, yet evidence on the combined effect of simultaneous investments in infrastructure, human resource, equipment, and governance remains limited in low- and middle-income countries. This study evaluated the effect of a multi-component health system intervention on productivity of eye service in seven Kenyan counties.

**Methods:**

A before-and-after study was conducted using longitudinal program data from the Vision Impact Project (2021–2024), a partnership between Christian Blind Mission (CBM), Kenya's Ministry of Health, county governments, and implementing partners. The intervention comprised: eye unit establishment; training of core eye health workers; provision of ophthalmic equipment; and establishment of county eye health technical working groups. Data sources included pre-implementation situation analysis (2019), project monitoring data, Kenya Health Information System, Kenya national population census data, and structured facility surveys.

**Results:**

The number of facilities, providing eye care services, increased from 5 to 27 across seven counties between 2019 and 2024. The training of 72 core eye health workers addressed 48% of the total workforce deficit at baseline across counties. Of 53 purposively selected high-value equipment, 50 (94.0%) were fully operational, 49 (92.0%) were routinely used, and 33 (62.0%) were first acquisitions. 7 out of 8 secondary level facilities reported reduced patient referrals to higher-level facilities. Five counties showed increased Cataract Surgical Rates (CSR). Each county established a functional eye health technical working group (TWG), with the inclusion of county eye health coordinators into County Health Management Committees (CHMCs).

**Discussion:**

Concurrent investment in facility infrastructure, human resources, equipment, and governance strengthening was associated with improved productivity of services in most of the counties. Variability in outcomes underscores that health system strengthening efforts should be intensified to broader health system constraints to achieve universal eye health coverage in Kenya.

## 1 Introduction

Health systems are interconnected structures in which human resource, governance, financing, service delivery, and medical technology jointly determine performance and outcomes (1, 2). The World Health Organization (WHO) framework for health system strengthening emphasises that progress towards universal health coverage requires simultaneous attention to all these building blocks, as weakness in any one area can undermine effectiveness of investments in others (1).

In health systems, productivity refers to the efficiency with which inputs such as human resources, infrastructure, equipment, governance structures) are transformed into outputs (services delivered, patients treated) (3). Within eye health services, productivity can be measured through indicators including service volume, population coverage rates such as cataract surgical rate (CSR), and utilization of available resources such as equipment and personnel (4). Higher productivity implies that more health services are delivered per unit of input, contributing to improved population health outcomes.

Globally, an estimated 2.2 billion people live with vision impairment or blindness, of whom at least 1 billion have conditions that are preventable or have yet to be addressed (5). The World Report on Vision (2019) calls for integrated people-centred eye care as part of universal health coverage, recognizing that eye health cannot be achieved through isolated interventions but requires strengthening of entire health systems (5).

In sub-Saharan Africa, the burden of avoidable blindness remains disproportionately high, with 5 million blind and 20 million moderately or severely visually impaired individuals (6). Despite this need, less than 1% of the world's ophthalmologists practice in the region, with only 13 countries meeting the minimum requirement of one eye health professional per 55,000 people (5). Setting of evidence-based targets for human resource for eye health (HReH) in the region remains a critical challenge (7).

Within ophthalmology specifically, the availability of both skilled health workers and functional diagnostic and surgical equipment is critical for accurate diagnosis, timely treatment, and effective disease monitoring (8). However, significant gaps in workforce availability and equipment functionality persist in low- and middle-income countries (LMICs), undermining efforts to reduce avoidable blindness (9, 10).

Parallel to workforce constraints, equipment deficits pose major barriers to quality eye care in LMICs (11). High procurement costs pose substantial barriers in resource-limited settings (11), and even when equipment is donated or purchased, limited local technical capacity for maintenance, lack of spare parts, and inadequate operator training frequently result in underutilization or premature failure (12, 13). Studies across LMICs consistently show that equipment non-functionality or underutilization stems less from the technology itself than from systemic deficits including lack of spare parts, unreliable infrastructure, and insufficient trained health workers (14, 15).

A fundamental prerequisite for service delivery is the presence of functional health facilities with dedicated space and infrastructure for eye care (16). The World Health Organization (WHO) identifies medical devices as integral to Health Systems Strengthening, with evidence demonstrating that appropriate technology deployment improves service quality, clinical outcomes, and system responsiveness (16, 17). However, procurement alone is insufficient; simultaneous investment in human resource, facility infrastructure, governance structures, and maintenance systems is essential (18). Ethiopia's experiences under the VISION 2020 “Right to Sight” global initiative illustrated that despite a comprehensive national strategy, gaps in human resource, infrastructure, and service integration limited progress toward targets (19).

In Kenya, an estimated 3.87 million people are affected by vision impairment, with over 80% of blindness attributable to preventable or curable causes (20). The National Eye Health Strategic Plan 2020–2025 identifies equipment deficits, maintenance backlogs, insufficient low vision aids, workforce shortages, inadequate infrastructure, and weak governance as major barriers to quality eye care (20). According to this Strategic Plan, there is a weak link between the National level Eye Health Inter-Agency Coordinating Committee and county governments which is the implementation level. There is also inadequate coordination of partner activities, especially at county level and a limited number of County Prevention of Blindness Working Groups. These challenges are compounded by maldistribution of qualified health workers, creating stark urban-rural disparities in service access (20). These factors result in diagnostic delays, treatment errors, avoidable vision impairment, and health worker frustration, disproportionately affecting rural marginalized populations (21, 22).

A Rapid Assessment of Avoidable Blindness (RAAB) survey data from 2024 indicated that cataract, glaucoma, and refractive errors are the leading causes of blindness in Kenya (23). Moreover, treatment coverage is low with only 35%–54% of those needing cataract surgery receiving it, and just 11% of individuals requiring corrective glasses access them (23). Barriers to provision of services include low community awareness, cultural misconceptions, out-of-pocket costs, geographical distance, and health system inefficiencies (21, 24).

The Vision Impact Project (VIP) was launched in 2021 as a partnership between CBM, Kenya's Ministry of Health, seven county governments (Bomet, Embu, Kiambu, Kajiado, Kwale, Meru, and Vihiga), and other implementing partners (25). The project was designed to complement national efforts towards universal eye health coverage. The aim was to strengthen eye care service delivery by addressing systemic gaps through concurrent investments in facility establishment and refurbishment, human resource training, ophthalmic equipment provision, and governance strengthening. This study aimed to evaluate the effect of this multi-component health system strengthening intervention on productivity of eye care services.

## 2 Methods

### 2.1 Study design

This is a before-and-after study without a control group conducted between 2021 and 2024. The baseline situation analysis was conducted in 2019, with project implementation commencing in 2021. This 18-month interval was primarily attributable to delays caused by the COVID-19 pandemic, which affected project planning, stakeholder engagement, and supply chain logistics. During this interval, counties continued routine service delivery, and some may have experienced changes in health workforce deployment, equipment status, or service volumes independent of the project. These baseline data therefore reflect the pre-intervention status of eye health services, and any changes observed between 2019 and 2024 represent the cumulative effect of both project activities and other concurrent factors.

### 2.2 Study setting

The study was conducted in seven counties in Kenya: Bomet, Embu, Kiambu, Kajiado, Kwale, Meru, and Vihiga. These counties were purposively selected for inclusion in the Vision Impact Project based on their high burden of avoidable blindness, existing health system partnerships, and commitment from county governments to strengthen eye care services. The counties represent diverse geographic regions and include both rural and peri-urban populations.

### 2.2 Selection criteria

All the seven project counties were included in this evaluation. For the structured facility surveys, eight government facilities were purposively selected for in-depth assessment: the seven county referral hospitals (one per county) and one additional high-volume facility in one county. These facilities were selected because they received the largest number of high-value equipment units under the project, providing geographic representation across the seven counties and capturing the facilities with the greatest equipment investments. The selection of equipment was purposive, focusing on high-value units for evaluation, with classification following the WHO Medical Devices Information System (MeDevIS) framework (17).

### 2.3 Health system strengthening interventions

The Vision Impact Project comprised four interconnected health system strengthening components implemented between 2021 and 2024:

1. Facility infrastructure and eye units:
  Establishment and refurbishment of eye units at county and sub-county level hospitals, which included the creation of outpatient consultation rooms, operating theatres, and installation of equipment.
2. Human resource development:
  Training of core eye care health workers, including ophthalmic clinical officers, cataract surgeons, ophthalmic nurses, low vision therapists, biomedical engineers, and training of non-eye health primary health care workers on the Ophthalmic Skills Upgrade Course (OSUC).
3. Equipment provision:
  Provision of ophthalmic equipment to secondary-level health facilities. Equipment types included optical coherence tomography (OCT) units, fundus cameras, ultrasound biometers, surgical microscopes, vitrectors, YAG lasers, slit lamps, tonometers, visual field analysers, and autorefractor/keratometers among others.
4. Governance strengthening:
  On the recognition that health systems performance depends not only on inputs but also on the structures and processes that coordinate them, the project included governance strengthening activities. These interventions responded to gaps identified in the National Eye Health Strategic Plan 2020–2025 and the VIP situation analysis baseline findings, which identified weak linkages between the Eye Health Inter-Agency Coordinating Committee and county governments, inadequate coordination of partner activities at county level, and a limited number of County Prevention of Blindness Working Groups.

Governance strengthening activities included:

1. Establishment of county eye health technical working groups
2. Capacity building for county eye health coordinators through a training course on eye health management and governance
3. Regular multi-stakeholder meetings bringing together county health managers, implementing partners, and facility leads.
4. Joint support supervision visits to health facilities
5. Sub-county implementation committee meetings to coordinate service delivery at lower levels.

These governance structures facilitated multi-sectoral engagement and created platforms for advocacy, including efforts to secure county government funding for eye health.

### 2.4 Data sources

Multiple data sources were used for this evaluation (See Table 1).

**Table 1:** Data sources for the evaluation.

Source	Description
Pre-implementation situation analysis (2019) report	The situation analysis evaluated health system strengthening pillars in study counties. Data utilized for this study includes number of eye units; availability of eye health workers assessed against WHO-recommended workforce-to-population ratios; cataract surgical data, and governance structures
Project monitoring data (2021–2024)	Training outputs (cadre, number trained, deployment); equipment inventories; service delivery indicators (cataract surgeries), formation of technical working groups & updated count of eye units
Structured facility surveys (2024)	Assessment of selected high-value equipment units for functionality, utilization, operator coverage, and service impacts across eight facilities
Kenya Health Information System (KHIS) (2021–2024) reports	Routine service indicators including the number of facilities offering eye care, number of cataract surgeries and workforce counts. *Cataract surgery data were obtained from the Kenya Health Information System (KHIS), which captures routine service reports from all health facilities in each county, including both government and faith-based facilities. The data presented therefore represent the total reported cataract surgeries for each county
Kenya National Bureau of Statistics (2019)	2019 Kenya national population census data with annual growth projections

### 2.5 Variables and measures

Dependent variables are presented in Table 2. Independent variables are:

1. Number of facilities established and refurbished to provide eye care services.
2. HReH training inputs: number and cadre of core health workers trained.
3. Equipment provision: type, quantity, and distribution of ophthalmic equipment.
4. Governance strengthening activities: establishment of County eye health Technical Working Groups (TWGs) and county eye care coordinators trained.

**Table 2:** Dependent variables.

Dependent variable	Definition/calculation
Number of facilities providing eye care services	Change in number of resolute, equipped eye units providing routine services at county and sub-county hospitals
Number of eye health workers	Change in number of trained core health workers; workforce coverage (percentage of required positions filled)
Equipment functionality	Percentage of installed equipment fully operational at time of assessment
Equipment utilization	Percentage of functional equipment in routine clinical use
Governance structures	Number of functional county eye health technical working groups (TWGs) established; number of county eye health coordinators trained
Referrals	Number of referrals to higher-level facilities
Cataract Surgical Rate (CSR)	Number of cataract surgeries per million population per year

### 2.6 Data analysis

Quantitative data were analysed using Power BI (version 2.131.901.0) for data aggregation, transformation, and visualization, complemented by Microsoft Excel for statistical calculations. Descriptive statistics summarized baseline eye units, workforce gaps, training outputs, equipment functionality, utilization patterns, operator coverage, and governance structures established. Percentage changes between baseline and endline were calculated for service indicators. Cataract surgical rates were computed using population estimates with annual growth projections. Facility survey data were analysed using descriptive statistics to summarize equipment functionality, utilization patterns, operator coverage, and service impacts. Qualitative data from open-ended survey questions and routine monitoring reports were analysed thematically to identify recurring themes related to barriers and facilitators to effective service delivery. These themes were triangulated with quantitative findings to provide contextual understanding of observed patterns. Facility and equipment data were collected and analysed using the structured facility survey instruments (See Supplementary File 1).

### 2.7 Ethics approval and consent to participate

This study forms part of a larger mixed-methods study titled “An Assessment of the Technical Efficiency of Eye Health Systems in Selected Counties in Kenya,” which received ethical approval from the Strathmore University Institutional Ethics Review Committee (SU-ISERC3066/25) and the National Commission for Science, Technology and Innovation (NACOSTI) (NACOSTI/P/25/4182006). As this study involved secondary analysis of program monitoring data and health system-level indicators, no individual patient data were accessed or analysed. All data were aggregated at facility or county level. As the secondary analysis did not involve interaction with human subjects or access to confidential personal data, the requirement for additional informed consent was waived.

## 3 Results

### 3.1 Change in the number of facilities providing eye care services

The change in the number of facilities providing eye care services in the project area between 2019 and 2024 as shown in Table 3. The total number of facilities providing eye care services across the seven counties increased from 5 to 27. Meru County had the largest expansion, growing from 1 to 10 facilities. Two of the counties (Vihiga and Kwale) had no facilities providing eye care services at baseline but had 1 and 2 units respectively by 2024.

**Table 3:** Change in number of facilities providing eye care across the seven counties between 2019 and 2024.

County	Eye units (2019)	Eye units (2024)	Absolute increase
Bomet	1	4	3
Embu	1	3	2
Kajiado	1	3	2
Kiambu	1	4	3
Kwale	0	2	2
Meru	1	10	9
Vihiga	0	1	1
Total	5	27	22

At baseline, two counties (Vihiga and Kwale) did not have government eye care facilities.

### 3.2 Change in number of skilled eye health workers

The baseline status of eye health workforce is shown in Table 4. Eye health workers were employed to staff the eight-participating secondary-level health facilities and selected sub-county hospitals within the project area with a mean of 8 workers per facility (range 4–11 workers). All facilities had dedicated health equipment maintenance staff, and all reported established equipment maintenance systems following the intervention.

**Table 4:** Baseline workforce requirements, availability, deficits, and training outputs by county.

County	Core health workers required *(n)*	Core health workers available *(n)*	Deficit (*n*, %)	Cataract surgeons trained *(n)*	Ophthalmic nurses trained	Biomedical technicians	Low vision therapists	Health workers trained in OSUC *(n)*
Kajiado	36	11	25 (69)	2	2	1	1	13
Embu	20	9	11 (55)	3	5	2	1	10
Meru	50	7	43 (86)	2	5	10	1	16
Bomet	29	16	13 (45)	4	8	1	3	27
Kiambu	77	45	32 (42)	1	2	1	0	13
Vihiga	20	12	8 (40)	2	5	1	1	35
Kwale	29	11	18 (62)	1	5	1	1	6
Total	**261**	**111**	**150 (57)**	**15**	**32**	**17**	**8**	**120**

OSUC, Ophthalmic Skills Upgrade Course.

The bold values: Sum of respective workers listed on the table.

At baseline, the total number of core eye health workers required across the seven counties was 261 and the deficit was 150 workers (57.5% gap). An additional 72 workers were trained, and this addressed 48.0% of the total deficit, and 27.5% of the total requirement. This increased workforce coverage from 42.5% to 70.1%.

Meru had the highest deficit of eye care health workers with a gap of 43 workers (86.0%), followed by Kajiado with 25 workers (69.4%). Vihiga had the lowest deficit with a gap of 8 workers (40.0%). Kiambu, despite having the highest absolute number of health workers at baseline (45), still faced a 41.5% deficit relative to requirement.

The additional 72 trained core eye care workers were distributed as follows: 15 ophthalmic clinical officer cataract surgeons, 32 ophthalmic nurses, 17 biomedical engineers, 8 low vision therapists and 120 primary healthcare workers who undertook an Ophthalmic Skills Upgrade Course (OSUC).

### 3.3 Equipment functionality and utilization

Of the 53 high-value equipment units, 33 units (62.3%) represented first acquisitions, these are equipment that had never been available in these facilities, while 20 units (37.7%) supplemented existing stock (See Table 5). Facilities reported that first acquisitions enabled new service offerings and improved care quality, while added acquisitions addressed insufficiency, obsolescence, or irreparable failure of existing devices. Of the 53 equipment units, 50 (94.3%) were fully operational at the time of assessment. Three units (5.6%) were non-functional due to financial constraints for troubleshooting, supplier maintenance challenges, or installation errors.

**Table 5:** Change in the number of total equipment in the seven counties.

Equipment	(*n*) before	(*n*) after	(*n*) additional	Observed
Tonometer	1	29	28	4
Autorefractor/Keratometer	5	9	7	7
Slit-Lamp	20	32	12	10
Optical Coherence Tomography (OCT)	0	3	3	3
Biometer, ultrasound	2	11	9	5
Ophthalmic Fundus Camera	0	6	6	5
Operating Microscope	14	24	10	8
Perimetry	5	8	3	3
Vitrector	1	9	8	5
YAG Laser Machine	0	3	3	3

Fifty (94.3%) were fully operational, and 49 (92.9%) were in routine use. The average operational period was 14 months, with 23 (43.4%) of devices having been in use for 13–15 months. Only one device (a YAG laser) was reported as underutilized due to training deficits.

### 3.4 Frequency of use

Twenty-three (43.4%) equipment were used daily, while 30 (56.6%) were used less frequently, often weekly. Qualitative data from facility surveys and routine monitoring revealed that infrequent use was associated with scheduled clinic days and limited operator coverage, particularly for specialized devices.

### 3.5 Changes in delivery of eye care services

Routine project monitoring data in all eight facilities reported service improvements following equipment provision. They revealed that before the equipment was provided, many patients with conditions such as glaucoma suspects were routinely referred to tertiary hospitals. Following the installation of OCT and fundus cameras, facilities were able to diagnose and manage these patients locally, which saved patients’ transport costs and time. However, some data noted that for certain specialized equipment like YAG lasers, there was limited number of trained operators which meant that patients were referred when a trained operator was not available.

Of the 8 facilities, 3 reported an increased scope of services and increased number of patients. Critically, 7 facilities reported reduced patient referrals to higher-level facilities, indicating strengthened local diagnostic and surgical capacity. Referral reduction was attributed to improved diagnostic capacity (OCT, fundus cameras) and surgical capability, enabling conditions such as glaucoma and diabetic retinopathy to be managed locally.

### 3.6 Staff satisfaction and workplace efficiency

Staff satisfaction and workplace efficiency were assessed though structured facility surveys conducted across all eight facilities. The leading survey question asked to what extent the provided equipment improved employee satisfaction, followed by a question on specific aspects of improvement. Responses were analysed descriptively and qualitatively. All eight facilities (100%) reported increased staff motivation and perceived better outcomes following equipment provision. Improvements related to workplace efficiency included enhanced diagnosis quality in five facilities (62.5%) and increased skill utilization in three facilities (37.5%). One facility (12.5) also reported improved collaboration with sub-specialists.

### 3.7 Cataract surgical rates

The relatively high cataract surgical activity observed in Vihiga County at baseline and endline warrants explanation. While Vihiga had no government eye units at baseline, cataract surgeries were performed through a faith-based facility and aggressive outreach services, which collectively accounted for the surgical volume reported. The project established one government eye unit in 2024, which will complement existing services. This underscores the importance of pluralistic service delivery models in achieving population coverage.

Table 6 presents cataract surgical rates (CSR) for the seven counties, comparing 2019 to 2024. CSR is presented alongside population figures to account for population growth when assessing changes in surgical productivity. Kiambu is the most populous county (2.42 million in 2019, projected 2.70 million in 2024). All counties experienced population growth ranging from 6.5% to 16.1%. CSR improved in five of seven counties, with Vihiga achieving the highest rate at 4,625 surgeries per million population. Kiambu's CSR declined from 1,147 to 901 per million population-adjusted output. Despite Embu's 502% increase in absolute cataract numbers, its CSR of 478 remains relatively low compared to other counties. Kajiado had the highest population growth rate (16.1%) and showed strong CSR improvement, increasing from 322 to 713. Five out of the seven counties had increased cataract numbers. Kiambu and Vihiga experienced declines (12.1% and 1.9% respectively). Though Vihiga and Kwale had no functional government eye units at baseline, cataract surgeries were still performed in these counties through faith-based facilities, private facilities, and outreach services.

**Table 6:** Cataract surgical rates.

County	Population estimates			Number of cataract surgeries done			CSR		
	2019	2024	Growth rate	2019	2024	% change	2019	2024	% change
Kajiado	1,117,840	1,298,095	16.1%	360	925	+156.9%	322	713	121.4%
Embu	608,599	655,057	7.6%	52	313	+502.0%	85	478	462.4%
Meru	1,545,714	1,646,169	6.5%	1,119	1,639	+46.5%	724	996	37.6%
Bomet	875,689	952,502	8.8%	1,609	1,972	+22.6%	1,837	2,071	12.7%
Kiambu	2,417,735	2,703,510	11.8%	2,774	2,437	−12.1%	1,147	901	−21.4%
Vihiga	590,013	631,045	7.0%	2,974	2,918	−1.9%	5,040	4,625	−8.2%
Kwale	866,820	966,260	11.5%	1,635	1,930	+18.0%	1,886	1,998	5.9%

Population projections for 2024 are based on 2019 Kenya national population census figures with annual growth rates applied.

### 3.8 Strengthened governance structures and county ownership

Prior to the intervention, governance mechanisms for eye health were weak across the seven counties. There was limited coordination between national and county levels, inadequate partner alignment, and few functional oversight structures. The baseline situation analysis, consistent with the National Eye Health Strategic Plan 2020–2025, identified weak linkages between the Eye Health Inter-Agency Coordinating Committee and county governments, inadequate coordination of partner activities at county level, and a limited number of County Prevention of Blindness Working Groups.

Following project implementation, each county established a functional eye health technical working group that met regularly to coordinate activities, review progress, and address challenges. These groups brought together county health managers, implementing partners, facility leads, and other stakeholders, creating formal platforms for coordination and accountability.

Stakeholder meetings brought together county health management teams, facility in-charges, and implementing partners, improving alignment of partner activities with county priorities. Joint support supervision visits were conducted, enabling coordinated problem-solving at facility level. Sub-county implementation committee meetings extended coordination to lower administrative levels, facilitating integration of eye health into broader primary care planning.

Capacity building for county eye health coordinators through a training course on eye health management and governance equipped them with skills in management, planning, budgeting, advocacy, and stakeholder coordination. As eye health became more visible in counties through advocacy efforts of eye health coordinators and implementing partners, counties lobbied for eye health funding from county governments. Some counties reported success in securing budget allocations for eye health activities, though the specific amounts and sustainability of these allocations require further tracking.

Prior to the project, partner activities were reported often fragmented and uncoordinated, leading to duplication or gaps in service coverage. The establishment of county technical working groups created a platform for partners to align their investments with county priorities and coordinate implementation schedules. County eye health coordinators reported that the management and governance training enhanced their confidence in engaging with county health leadership and advocating for eye health alongside other competing priorities. Some coordinators noted that the regular stakeholder meetings improved relationships between county health departments and implementing partners, fostering greater trust and collaboration.

### 3.9 Barriers and facilitators to effective service delivery

The facility surveys and routine monitoring data also revealed several themes explaining the quantitative findings:

Availability of equipment increases clinical confidence and patient trust. The clinicians commented that the ability to show patients images of their retina using fundus cameras improved patient understanding of their conditions and increased trust in treatment decisions. Clinicians also reported that this visual confirmation reduced patient default rates and improved adherence to follow-up appointments.

Maintenance systems require ongoing attention. Facilities also reported equipment downtime due to delayed procurement of spare parts, with one microscope non-functional for three months while awaiting a part to be imported from overseas. Facilities that maintained spare part inventories experienced shorter downtime, but budget constraints limited the ability to stock all necessary components. Biomedical engineers emphasized the need for reliable supply chains and dedicated maintenance budgets.

It was also observed that the establishment of county technical working groups created platforms for partners to align investments with county priorities which strengthened the governance and improved coordination and advocacy. Some counties successfully lobbied for eye health budget allocations, demonstrating the tangible impact of strengthened governance.

Governance structures strengthened coordination and advocacy. The establishment of county technical working groups created platforms for partners to align investments with county priorities, which strengthened governance and improved coordination and advocacy. Some counties successfully lobbied for eye health budget allocations, demonstrating the tangible impact of strengthened governance.

Infrastructure expansion enables service decentralization. The establishment of eye units in sub-county hospitals reduced travel distances for patients seeking basic eye care. Clinicians reported that this decentralization allowed screening and treatment of common conditions at local facilities, with only complex cases requiring referral to higher levels. This improved geographic access and reduced the burden on central referral facilities.

## 4 Discussion

The findings of this study demonstrate that concurrent investment in facility infrastructure, human resource, ophthalmic equipment, and governance was associated with substantial improvements in service delivery indicators. The observed changes suggest that an integrated approach to health system strengthening can yield measurable improvements to service delivery. However, the variability in outcomes across counties reveals important lessons for understanding how context shapes the effectiveness of such investments and highlights areas requiring sustained attention.

### 4.1 Infrastructure for eye care service delivery

The expansion of facilities providing eye care services represented the most foundational achievement of the intervention. The Kenya National Eye Health Strategic Plan states that without dedicated consultation space, appropriate lighting, reliable power and water, and properly configured examination rooms, even the most sophisticated equipment and best-trained health workers cannot function effectively (20). Moreover, Allen L et al. observed that establishment of dedicated space for eye care services at sub-county level brings services closer to communities, reducing travel distances and associated costs for rural populations (21).

The study findings indicated that infrastructure expansion enabled service decentralization, with clinicians reporting that patients no longer needed to travel long distances for basic eye care and could be diagnosed and treated at their local hospitals. This aligns with evidence from the LV Prasad Eye Institute's VISION 2020 model in India, which demonstrated how a networked approach with primary, secondary, and tertiary facilities can significantly improve service delivery outcomes and population coverage (26).

### 4.2 Combined benefits of integrated health system strengthening efforts

The equipment high functionality and utilization rates reported in this study contrasted with earlier studies from Kenya, where diagnostic equipment was often absent or underused (27). This underscores the fact that supply alone is insufficient without concurrent system support. The VIP model's integration of functional infrastructure, maintenance systems, training, and governance contributed to the high functionality and utilization rates observed. This aligns with evidence from other reports from the African region that equipment investment must be paired with systematic workforce development and maintenance infrastructure (18, 28).

The finding that the majority of the evaluated equipment units were first acquisitions highlights significant baseline deficits in diagnostic and surgical capacity across facilities. Introduction of advanced technologies such as biometers, perimeters, and OCTs enabled services previously unavailable, expanding clinical scope and diagnostic reach. These findings agree with other studies conducted in African settings by Du Toit and Maro F who reported that bridging fundamental technology gaps is pivotal to health system strengthening in resource-limited settings (29, 30).

The reduction in patient referrals to higher-level facilities reported in most secondary level facilities represents a crucial gain in a context where tertiary facilities are overburdened and geographically inaccessible to many patients (23). This finding aligns with evidence from other sub-Saharan African settings demonstrating that decentralized diagnostic capacity improves case detection, reduces delays, and enhances health system efficiency (31, 32). The reduction in referrals suggests that equipping secondary-level facilities can alleviate pressure on tertiary facilities while enabling earlier intervention closer to patients' communities.

### 4.3 Governance for sustained health system strengthening

The governance strengthening activities under VIP addressed critical coordination and oversight gaps identified at baseline. The establishment of county eye health technical working groups in each county created formal structures for partner coordination, reducing duplication and ensuring alignment with county priorities (33). This finding aligns with evidence that effective health system governance requires platforms for multi-stakeholder engagement and clear accountability mechanisms (3).

Prior to the intervention, partner activities were often fragmented, with limited coordination between the national Eye Health Inter-Agency Coordinating Committee and county governments. The county-level technical working groups bridged this gap, creating regular forums for county health managers, implementing partners, and facility leads to review progress, to address challenges, and coordinate implementation. Joint support supervision visits further strengthened accountability by bringing multiple stakeholders together for facility-level problem solving.

The capacity building for county eye health coordinators was particularly significant, as these individuals serve as the link between national policy and county implementation. Strengthening their skills in planning, budgeting, advocacy, and stakeholder coordination positioned them to champion eye health within the county. The reported success in securing additional county government funding, while variable, suggests that governance investments can translate into sustained domestic financing, a critical consideration for long-term sustainability beyond project timelines (34).

However, as Tsofa et al. (35) observed in their analysis of Kenyan county health systems, variability in funding outcomes across counties often reflects differences in pre-existing administrative capacity and political will. This underscores that governance strengthening should be an ongoing process rather than a onetime intervention.

### 4.4 Human resource development for productivity gains

The training of core health workers addressed a substantial portion of the baseline workforce deficit. The project's focus on core eye care cadres of cataract surgeons, ophthalmic clinical officers, ophthalmic nurses, biomedical engineers, and low vision therapists reflects recognition that specialist health workers are essential for delivering advanced services. This approach aligns with evidence linking workforce development to improved service delivery in resource-constrained settings (18, 36).

Staff morale improvements align with evidence linking equipment adequacy and training to workforce motivation and retention in resource-constrained settings (18, 36). Enhanced clinician-patient interactions and increased trust facilitated by diagnostic imaging, are consistently associated with improved service uptake and outcomes (37).

### 4.5 Variability in cataract surgical rates: understanding contextual factors

The variable patterns of cataract surgery changes across counties highlight the importance of context in shaping health system strengthening outcomes. Kiambu County requires examination of multiple interacting factors. Its experience illustrates how proximity to a major urban centre with concentrated specialist services can fundamentally alter patient utilization patterns. This is in line with evidence from other studies (38–40) indicating that higher cataract surgical coverage is associated with the presence of at least one fixed surgical facility, access to a dedicated operating theatre, and a greater density of surgeons per million population. Such patterns have been widely documented in the literature, reinforcing the role of health system capacity and geographic accessibility in determining surgical uptake (41).

Our interpretation of Kiambu's declining CSR is based on three lines of evidence. First, the Kenya Health Information System data capture only surgeries performed within county government and reporting faith-based facilities; patients from Kiambu who seek services in Nairobi would not be captured, potentially underestimating actual surgical coverage for Kiambu residents. Second, qualitative data from facility surveys revealed that patients commonly bypass Kiambu county facilities in favour of the higher concentration of specialist services in Nairobi, consistent with urban health market dynamics documented elsewhere (41). Third, population growth analysis shows that even maintaining the same absolute surgical volume would have resulted in declining CSR; the observed 12.1% drop in absolute surgical volume created a double burden of fewer surgeries for a larger population. This interpretation is supported by literature from Kenya demonstrating that bypassing of government facilities occurs when patients perceive tertiary or private facilities to offer higher quality care, irrespective of distance travelled (41). While acknowledging that these are plausible explanations rather than confirmed causal mechanisms, triangulation across multiple data sources strengthens this interpretation.

Vihiga county presents a different pattern, demonstrating that high surgical productivity is achievable. The sustained high performance, driven by a combination of faith based and government sector contributions suggest that pluralistic service delivery models can collectively address population need. This pattern is in line with findings from Kenya that demonstrates that pluralistic delivery models in which government facilities and faith-based organizations function in complementary roles within devolved health systems achieve high and sustained surgical productivity (42). Faith based organizations make up a significant share of Kenya's hospital capacity, especially in rural areas, and they exhibit service readiness and management quality levels that are on par with, and occasionally higher than those of government hospitals (42).

Embu's high CSR increase should be interpreted alongside its extremely low baseline. While this was a significant improvement, it remains relatively low compared to other counties. This illustrates that percentage changes can be misleading when baselines are incredibly low, and absolute gains must be assessed against population need.

### 4.6 Implications for eye health systems strengthening

These findings carry several implications for policy and practice. Firstly, health system strengthening requires integrated, multi-component approaches. The VIP model's success in expanding eye units, achieving high functionality and utilization rates, and establishing governance structures demonstrates that infrastructure, equipment, human resource, and governance investments must proceed together. Programs seeking to scale eye care services should adopt adaptive management to address emerging constraints across all health system building blocks.

Secondly, workforce planning must be context specific. Counties require tailored strategies based on local epidemiology, existing workforce composition, infrastructure, population distribution, and health market dynamics.

Thirdly, eye units are the foundational platform for eye care service delivery. County governments should prioritize establishing functional units at sub-county level to improve geographic access and reduce referral burdens on tertiary facilities.

Fourthly, equipment deployment plans must include explicit training targets, and training strategies should be tailored to equipment complexity and anticipated utilization frequency. In addition, equipment maintenance systems require dedicated funding and local capacity.

Lastly, governance structures are essential for coordination and sustainability. The establishment of county eye health technical working groups created platforms for partner coordination, alignment with county priorities, and advocacy for domestic financing. Strengthening these structures at county level is essential for sustaining gains from other health system inputs.

## 5 Limitations

This study had several limitations. Firstly, the before-and-after design without a control group limits causal inference, as the observed changes may be influenced by external factors including broader health sector trends, or other development partner activities.

Secondly, the 18-month interval between the baseline assessment (2019) and implementation start (2021) introduces the possibility that changes in health system context such as workforce turnover or other partner activities may have influenced baseline-to-endline comparisons independent of the project intervention. While we have attempted to account for these through multiple data sources and qualitative exploration of contextual factors, this remains a limitation of the study design.

Thirdly, the 14-month observation period for equipment outcomes may not capture long-term sustainability challenges, particularly regarding maintenance and workforce retention. Equipment functionality often declines over time as devices age and maintenance challenges accumulate.

Fourthly, reliance on routine project and health system data may be subject to reporting variability, inconsistencies in data quality, and changes in reporting systems over time.

Lastly, the analysis did not assess service quality or patient-level outcomes such as visual acuity improvement after cataract surgery. Future research should examine whether increased surgical volume translates into maintained or improved quality.

## 6 Conclusions

This evaluation of the Vision Impact Project in Kenya demonstrated that concurrent investment in human resource, ophthalmic equipment, governance, and facility infrastructure was associated with improved eye service productivity across most participating counties. These findings support integrated health system strengthening approaches that simultaneously address infrastructure, human resource, equipment, and governance to achieve universal eye health coverage in Kenya and similar settings. Future research should examine the long-term sustainability of these investments, the durability of governance structures after project conclusion, optimal training models for mid-level cadres operating specialized equipment, cost-effective maintenance strategies, and patient-level outcomes including visual acuity improvement and quality of life.

## Data Availability

The raw data supporting the conclusions of this article will be made available by the authors, without undue reservation.
